# Healthy helpers: using culinary lessons to improve children’s culinary literacy and self-efficacy to cook

**DOI:** 10.3389/fpubh.2023.1156716

**Published:** 2023-11-06

**Authors:** Peggy Policastro, Alison H. Brown, Erin Comollo

**Affiliations:** New Jersey Institute for Food, Nutrition, and Health, New Jersey Healthy Kids Initiative, Rutgers University, New Brunswick, NJ, United States

**Keywords:** self-efficacy, culinary literacy, vegetable intake, cooking skills, children, culinary knowledge, recipe acceptance, willingness to eat vegetables

## Abstract

**Background:**

Children do not eat the recommended amounts of vegetables, and school-based nutrition education has not been found to impact this behavior. Cooking education is associated with improved children’s culinary literacy (CL) and eating behaviors. This study investigated the impact of a culinary literacy (CL) curriculum on children’s acceptance of vegetable-added (mushrooms) recipes, CL, self-efficacy to cook (SE), and willingness to try vegetables (WV).

**Methods:**

A convenience sample of 39 fourth and fifth graders were exposed to a six-lesson virtual CL curriculum that taught basic cooking skills and how to prepare six recipes, including three traditional recipes and the same recipes with added vegetables.

**Results:**

Children who participated in the CL curriculum accepted vegetables added to pizza pockets, but vegetables added to macaroni and cheese and fajitas negatively affected the acceptance of recipes. Children improved their CL and SE but did not show a significant change in their WV.

**Conclusion:**

Findings suggest that CL programs focusing on vegetables may drive factors associated with dietary behavior change, SE, and acceptance of vegetables. Future studies should consider CL as a potential method to improve vegetable intake in children in tandem with nutrition education or as a sole intervention. The study was limited by its small sample size, the virtual setting, and the use of mushrooms as the primary vegetable source. Future studies should be conducted with a larger sample size, in a traditional classroom setting, use a variety of vegetables, and collect qualitative data on the sensory characteristics affecting children’s WV.

## 1. Introduction

Childhood is a critical time for establishing healthy lifelong eating habits and dietary behaviors that often continue into adolescence and adulthood (1). A well-balanced diet containing adequate amounts and varieties of fruits and vegetables is necessary for children to maintain appropriate caloric intake and body weight, support nutrition adequacy, and reduce the risk of chronic disease (2). Despite the established health benefits, children continue to fall short of the daily recommended intake of 2–4 cups of total fruits and vegetables, as outlined in the Dietary Guidelines for Americans 2020–2025 (3, 4). Suboptimal intakes in children predict poor fruit and vegetable intakes as they age and progress into adolescence and adulthood (2, 4, 5). The disproportionately substandard produce intake among lower-income families is even more troubling, as is lower intakes of vegetables when compared to fruit (5, 6). Previous research on children’s fruit and vegetable intakes and interventional programs have been broad and did not focus solely on vegetables or explore the role cooking education and experiences may have on culinary literacy, self-efficacy to cook and children’s acceptance and willingness to try recipes with vegetables, the focus of this study.

To successfully increase children’s vegetable intake, it is important to address barriers that impact access and acceptance of this food group. Two barriers to children’s acceptance of vegetables are their unfamiliarity to the child and dislike of how they taste (5, 7, 8). The high cost of vegetables is an added familial barrier (5, 9). School-based programs have been tasked to address these barriers, with most focusing on educating children and families about health benefits and/or providing free/subsidized fruits and vegetables. However, awareness of health benefits does not address the acceptance of produce, and programs have reported no significant effects on vegetable intakes (6). Research has suggested that school programs may be more effective by directly addressing children’s familiarity to vegetables and their taste (8, 10). Previous research has achieved this by providing children with regular small tastings, hiding vegetables in meals, or serving them in an identifiable form (8, 10, 11). Another method to employ these strategies of familiarizing children to vegetables in the school environment could be to create an intervention centered around promoting culinary literacy (CL), the set of abilities that allow individuals to prepare meals from scratch.

Cooking behaviors/frequency and CL among young children and families is an emerging area of study with the potential to improve eating behaviors, including the increased consumption of produce (11–13). Studies focusing on cooking behaviors find that with the decline in fruit and vegetable intake, there is also a concurrent decline in home cooking and CL levels among adults (12, 14, 15). The relationship between CL and nutrition is multigenerational. As home cooking declines, culinary knowledge, and skill development decrease among younger generations due to limited exposure to culinary processes and a lack of adult/peer modeling. This has resulted in low cooking collective efficacy among the entire family unit (12, 16). Higher CL and increased home cooking frequency have been linked to improved dietary quality as home-cooked meals have been found to naturally contain more fruits and vegetables compared to pre-prepared meals (13, 14). More interventions to promote vegetable intake among children must examine the connection between CL, improved cooking self-efficacy, and dietary behavior changes.

Interventions that feature experiential learning like cooking are unique in that they provide hands-on activities that require active participation and multisensory experiences (17, 18). Children learn about the world through their senses and interacting with vegetables may reduce the initial apprehension they might feel toward new foods. Children often hesitate to try new foods like vegetables, resulting in inadequate intake (19). They should interact with vegetables to reverse this trend. Involving children in the kitchen and having them prepare recipes that feature fresh produce provides direct contact, increased familiarity, and repeated taste exposures to vegetables. Research has reported that these factors have been related to increased self-selection of produce by children (11, 14). Offering experiential learning through cooking in the school environment provides knowledge with an accompanying skill set and may have a greater influence on their intake of vegetables (1, 11). Beyond their experiential nature, culinary education programs are social and allow for ownership and empowerment and have been linked to improved nutrition-related beliefs, self-efficacy, knowledge, and behaviors (15, 16). However, many research interventions that focused on child involvement in meal preparation were part of a multicomponent program or folded in other food literacy topics such as nutrition education and gardening. The multicomponent aspect of these programs made it difficult to parse out which programmatic components led to behavior change. Lack of focus on CL behaviors may account for limited gains in knowledge and skills among participants (18, 20, 21). This study is novel as the singular focus is on CL behaviors as drivers of behavior and self-efficacy change that may result in willingness to try vegetables.

Previous efforts to increase vegetable intake among children, particularly those within the school setting, have failed to elicit enhanced vegetable intake, and novel approaches are needed. This study examined the effect of a school-based CL program that featured at-home cooking experiences on children’s acceptance of vegetable-added recipes and willingness to try vegetables without direct nutrition education. The results of this study will add to the body of research emerging about best practices to encourage children from lower-income families to eat more vegetables, achieve recommended vegetable amounts, and improve the nutrition behavior drivers of culinary knowledge and self-confidence/efficacy in the life skill of cooking.

## 2. Materials and methods

### 2.1. Participants

Participants were a convenience sample of children in a health/physical education class at a Charter School in an urban area of New Jersey. Approximately 82% of children received free/reduced school lunches. Children of all genders and demographics were eligible to participate if they were in fourth or fifth grade, had written parental/guardian consent to participate in the study, had no medical or behavioral aversion to taste the foods, had access to a computer with video for Zoom® sessions and survey completion, and could speak or understand English or Spanish at a proficient level. The Rutgers University Institutional Review Board reviewed and approved the study protocol.

### 2.2. Description of the intervention program

The *Healthy Helpers: Culinary Literacy for Kids* research study used a 6-week curriculum to teach elementary-aged children CL, including basic cooking skills and concepts. The curriculum design was informed by the Social Cognitive Theory (SCT), which claims that behavior change results from factors such as self-efficacy and experiential learning. Lessons and activities were inspired by existing food literacy curricula, specifically Cooking Matters and In Defense of Food (21–24). The curriculum was developed and reviewed by a registered dietitian with a doctorate in nutrition psychology, a Registered Dietitian Nutritionist (RDN) specializing in community nutrition, a clinical RDN, and a former classroom educator with a doctorate in education. An RDN taught one weekly lesson, and the children’s physical education teacher supervised the classes. The original program design was to teach lessons during the regularly scheduled school day. Due to safety precautions surrounding the COVID-19 pandemic, lessons were revised to be taught virtually via the online platform Zoom®, and surveys were collected via the online survey tool, Qualtrics®. Lessons were taught, and data were collected during March and April 2021. Each lesson featured a cooking concept or skill followed by a recipe cooking demonstration. Cooking skills promoted cooking techniques and strategies for children to perform the skill independently, such as grating cheese using a box grater, using scissors to cut, safe knife handling, and using the microwave safely. Cooking demonstrations featured recipes in a traditional form familiar to the children or in a version that included mushrooms (Supplementary Table 1). Mushrooms were chosen as the added vegetable as they have the unique quality of taking on the flavor of the ingredients they are combined with, do not impart a bitter taste, are not a common food allergen, and are readily available in many forms, including fresh, frozen, and canned (22, 23). Recipes were randomized to limit order bias using a Latin square design. All recipes can be found in the Supplementary Material.

Participating children were provided weekly cooking kits at no cost to the family to encourage participation and decrease the economic burden of cooking activities. Each kit contained a printed copy of the weekly recipe and all the ingredients required for that week’s recipe. Children received their kits as part of their pre-existing weekly school meal pickups. Cooking kits did not contain kitchen tools since program recipes were designed only to require the equipment and tools commonly found across all socioeconomic households (microwave, measuring spoons, utensils, scissors, and a grater) (24). To ensure that families had what they needed to complete the cooking assignments, participants were told that they could request any missing pieces of kitchen equipment required for the recipes. No requests for additional equipment were made.

After viewing the weekly live lesson via Zoom®, children replicated that week’s cooking demonstration at home using the cooking kits independently or with familial support. Children had until the following week’s class to complete their home cooking assignment. After cooking the weekly recipe, children were asked to take a picture of their dish, taste the food product prepared, and complete the online Post Recipe Acceptability Survey (PRAS) with eight questions to assess the acceptability of the recipe. Children were sent regularly scheduled reminders by the RDN and physical education teacher via the existing Google Classroom site and were instructed to follow the recipe exactly as written. As backup instruction, a YouTube link to the cooking demonstration video shown in class was provided to each child for reference. The cooking demonstration videos and recipes were provided in English and Spanish for bilingual households.

### 2.3. Survey instruments, measures, procedures, and data analysis

**Figure 1:**
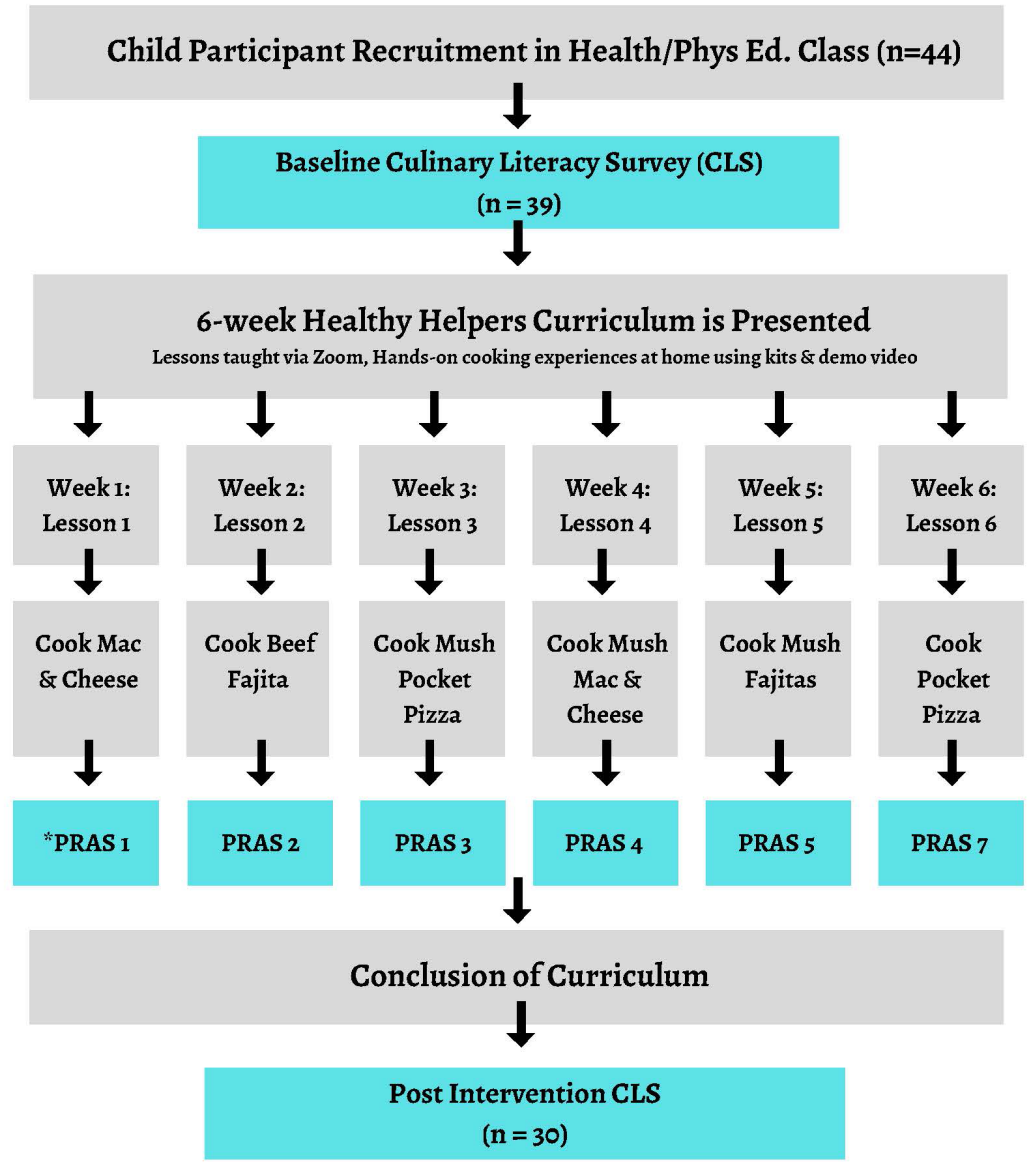
Sequence of participation in Healthy Helpers; Culinary Literacy for Kids Curriculum and Survey collection. PRAS, Post recipe acceptance survey.

We collected data via two validated survey that were informed by research and expert reviewed via the online survey platform Qualtrics®: the PRAs and the Culinary Literacy Survey (CLS). Each child completed the PRAS after completing each of the six at-home cooking assignments, and the CLS was completed by each child at baseline and post-program (Figure 1). All surveys are available in the Supplementary Material.

#### 2.3.1. Post recipe acceptability survey

An eight-item quantitative survey was administered to each child after they made and tasted each of the six recipes to assess the acceptance of each recipe. Acceptability was based on the child’s liking of the food, their desire to eat it again, their desire to cook the recipe again, and whether they would choose the food if it were offered at school lunch. Answers of “no” were coded as 1, and answers to “yes” were coded as 2. Therefore, the minimum acceptability score was 1, and the maximum score was 2. The coded responses to the Post recipe acceptability survey (PRAS) were aggregated and averaged into a total acceptability score for each of the six recipes tested.

#### 2.3.2. Culinary literacy survey

The Culinary literacy survey (CLS) was administered in a quasi-experimental, pre-post survey design to assess fourth and fifth grade children’s CL skills. Questions in the CLS were derived from the Tool for Food Literacy Assessment in Children (TFLAC) validated survey to assess culinary literacy knowledge, self-efficacy to perform basic cooking tasks, and overall willingness to eat vegetables (25).

#### 2.3.3. Variables and data analysis

*Post hoc* power analysis was conducted using SPSS, version 27, to test the difference between paired sample group means using a two-tailed test with a medium effect size (d = 0.50) and an alpha of 0.05. Results showed with the criteria stated above, our sample of participants would achieve a power of 0.80. Recipe acceptance scores were calculated by adding the answers to four questions and taking the mean score (Yes-2, No-1): Did you like the recipe? Would you eat it again? Would you make it at home if you had everything you need and the help of an adult? Would you eat this food if it was offered at school lunch? The maximum recipe acceptance score was 2, yes to all four questions, and the minimum recipe acceptance score was 1, no to all four questions. Paired t-tests were performed on data collected from the PRAS to determine if there was a significant statistical difference in recipe acceptance scores of traditional vs. vegetable-added recipes.

Quantitative data collected on the CLS were classified into three subcategories: (1) culinary literacy knowledge (CL); (2) self-efficacy to perform basic cooking tasks (SE); and (3) willingness to eat vegetables (WV). Child responses were coded with 0 indicating incorrect answers/negative responses and 100 indicating correct answers or the most positive responses. The minimum scores in each area were 0, and the maximum was 100. After calculating each child’s score, descriptive statistics were used to calculate the mean and standard deviation for each of the three variable subcategories. A total score for CLS was calculated by taking the mean of all three subcategory scores and the standard deviation of the total score mean was presented. We addressed missing data by imputing the series mean for 25% of the missing values. Paired *t*-tests were used to determine if there was a significant statistical difference in the means of pre- and post-scores on the CLS survey for the three dependent variables (CL, SE, WV) and total survey score.

## 3. Results

Children participating in the *Healthy Helpers: Culinary Literacy for Kids* program had improved outcome measures that may predict improved vegetable intakes among this population. Of the three recipes tested, children best accepted added vegetables as pocket pizzas. Added vegetables to macaroni and cheese and fajitas negatively influenced children’s acceptance of these recipes. Participation in the curriculum improved nutritional behavior drivers. These included significantly increasing children’s culinary self-efficacy and culinary literacy knowledge. We did not observe increased measures assessing children’s willingness to eat vegetables (Supplementary Table 4).

### 3.1. Participant demographics

Forty-four parents/guardians consented for their children to participate in this study. Of the consented children, 39 (89%) completed the CLS pre-intervention, and 30 (68%) completed the survey post-intervention. The mean age of the children was 10.6 (SD 0.6) years old, with the majority being female (59%). Most children were Hispanic (87%) and were receiving free and reduced breakfast/lunch (82%; Supplementary Table 2).

### 3.2. Child acceptance of traditional vs. added-vegetable recipes

To test the hypothesis that there would be no significant difference in children’s acceptance of recipes with and without vegetables, we examined paired *t*-tests for a significant statistical difference between recipe pairs, i.e., traditional recipes and the same recipe with added vegetables. There was no statistical difference between the children’s acceptance of the traditional and mushroom pocket pizza recipes [*t*(9)=0.712, 0.494]. We found a significant difference between the children’s liking of the traditional macaroni and cheese and the recipe with increased vegetables [mushroom macaroni and cheese; *t*(12)=3.600, 0.004]. We also found a significant difference in the children’s acceptance of the traditional beef fajita recipe and the fajita with added vegetables (portabella fajita) with children preferring the beef fajita [*t*(14)=2.739, 0.008]. The children’s liking of the pocket pizzas was not affected by adding vegetables; however, vegetables added to macaroni and cheese and fajitas negatively impacted the liking of these recipes (Supplementary Table 3).

### 3.3. Child culinary knowledge, self-efficacy to cook, and willingness to eat vegetables

To test the hypothesis that CL lessons would improve nutrition behavior drivers in fourth and fifth grade children, we calculated descriptive statistics of mean and standard deviation of pre- and post-CLS scores for each sub-category: children’s culinary literacy knowledge (CL), self-efficacy to cook (SE), and willingness to eat vegetables (WV) and for the total survey score (Supplementary Table 4). Paired *t*-tests were used to determine if there was a significant statistical difference between the means of pre- and post-CLS scores for each sub-category and the total survey score. Results of these analyses revealed a statistically significant increase in children’s pre-post self-efficacy to cook [*t*(38)= − 16.064, *p* < 0.001] and total scores [*t*(38)= −8.088, *p* < 0.001]. There was no significant difference in willingness to eat vegetables pre- and post-CL lessons [*t*(38)= −0.168, *p* = 0.437].

## 4. Discussion

Children are not consuming enough vegetables and are eating less home-cooked meals (26). This study aimed to determine the role of CL lessons and home cooking experiences on children’s acceptance of recipes with added vegetables and self-efficacy to prepare these recipes, without direct nutrition education. Results showed that *Healthy Helpers,* a school-based CL program that featured home cooking experiences, significantly enhanced children’s acceptance of vegetable-added recipes and improved children’s self-efficacy to cook independently (*p* < 0.05). *Healthy Helpers* was a novel approach to improving children’s self-efficacy in CL and acceptance of recipes with added vegetables. These results are consistent with previous research that has connected cooking with improved dietary behaviors (11, 16, 27).

The objectives of this study are unique in that the CL portion of food literacy was parsed out to examine CL factors (basic cooking skills, vocabulary, and safety) that may influence the acceptance of vegetables (mushrooms) in recipes among elementary-aged children from limited-resource families. Outcome measures included children’s liking of recipes featuring vegetables, likelihood to eat these foods again, intent to prepare the recipe at home, and likelihood to choose the food when offered on the school lunch menu. This study sought to achieve these objectives by combining pedagogical strategies such as adult and peer modeling to deliver lessons about CL via a digital platform (Zoom®). The results of this study will add to the body of research emerging about best practices to encourage children from lower-income families to eat more vegetables, achieve recommended vegetable amounts, and improve the nutrition behavior drivers of CL and self-confidence/efficacy in the life skill of cooking.

The first objective of this study was to determine if children would be more likely to accept added vegetables to recipes if they were involved in the cooking process. The recipe with added vegetables accepted by children the same as/equal to its traditional version was the mushroom pocket pizza. We propose two explanations for why vegetables may have been best accepted in this recipe. First, the pocket pizza recipe was unique because it was the only recipe to add vegetables (mushrooms) in a preparation that was not visible to the children; they were enclosed within a dough pouch. Second, the strong flavor combinations of marinara sauce, cheeses (mozzarella/parmesan), and spices (basil/oregano) may have masked the presence of the vegetables better than the other recipes. The method of “hiding vegetables” by adding them in ways that maintain the recipe’s appearance and overall flavor has been cited by other studies as an effective strategy to enhance vegetable intake among children and should be considered by parents, nutrition practitioners, and schools as a way to improve children’s acceptance of recipes containing vegetables (8, 28).

The vegetable chosen for the research design (mushrooms), may account for the children’s limited acceptance of the remaining added vegetable macaroni and cheese and fajitas recipes. Mushrooms are a vegetable that may not be readily accepted by some children. A study that analyzed children’s vegetable preferences in 2019 revealed that among six common vegetables tested (broccoli, corn, cucumber, mushrooms, potatoes, and sweet peas), mushrooms were the only vegetable rated as “dislike” in the United States (29). In this manner, all three of our recipes were disadvantaged since mushrooms were the vegetable added to all. The macaroni and cheese recipes and fajitas highlighted the mushroom’s presence more prominently/than the pocket pizza, which may have contributed to the recipe’s lower rating by the children. The questions within the PRAS did not allow us to determine the specific sensory properties that contributed to children’s dislike of the recipes, i.e., color, flavors, textures, aroma. Future studies may consider a mixed method model to examine qualitative factors and determine the sensory properties contributing to or detracting from children’s acceptance of vegetables.

The second and third objectives of this project were to measure the impact of a CL-focused program on nutrition behavior drivers in children, such as culinary literacy knowledge (CL), cooking self-efficacy (SE), and willingness to eat vegetables (WV). Lesson content and survey methods used in the project were specifically informed by a theoretical framework that explains how an individual learns, the Social Cognitive Theory (SCT). SCT posits that learning is affected by a blend of personal, interpersonal, and environmental factors that interact in a dynamic and ongoing process to influence behavior (26). These features are unique to nutrition education methods that rely on learning via skill-based approaches and are not present among didactic methods. Children may currently elicit low levels of CL due to the absence of experiential learning opportunities in cooking within the two places they learn most: school and home. The *Healthy Helpers: Culinary Literacy for Kids* curriculum was a novel approach to improving children’s vegetable intake by increasing their CL and cooking skills, as many school-based nutrition education interventions do not support skill development (30, 31). Children may have the didactic knowledge that vegetables are a healthful and important part of their diet but lack the “culinary tools” necessary to prepare them for themselves or offer cooking suggestions to their caregivers.

One of the key mechanisms described within the SCT is the concept of self-efficacy (SE), which is the belief by an individual that they possess the requisite cognitive abilities, motivation, and resources to complete the task (32). The SCT states that self-efficacy plays a significant role in motivating an individual to change behavior and is best developed by providing information via different channels, including mastery experiences, collective efficacy, and persuasion (33). Lessons were designed to contribute to children’s self-efficacy in cooking by including hands-on experience, skill mastery, problem-solving strategies, and social interaction (at home and within the virtual classroom).

According to SCT, childhood learning is a remarkably complex process highly influenced by social modeling and persuasion between peers and caregivers. *Healthy Helper’s* virtual lessons provided unique opportunities for social modeling in school and home environments, which play a critical role in determining how and what children learn. Peer modeling was incorporated within each lesson by having children share their thoughts during virtual class about culinary concepts and their cooking experiences, which built collective efficacy. Children also had a chance to show their finished dishes to each other by posting pictures of their recipes online. These pictures not only highlighted their accomplishments but also supported their development of self-regulation skills and fosters a sense of ownership, other components of the SCT. In addition, the cooking assignments provided adult/child modeling and persuasion within the home by encouraging caregivers to assist the children in making the recipes. The *Healthy Helpers* cooking experiences got children in the kitchen and encouraged families to prepare nutritious foods from scratch.

Home economics, a course that taught basic food preparation skills and meal planning, is no longer a fixture in school districts. To improve education about food, it may be necessary to bring back some of the concepts taught within a home economics course, such as those included in the *Healthy Helpers* curriculum (34). The *Healthy Helpers* cooking demonstrations and at-home cooking assignments advanced children’s behavioral capability to cook, which is another feature of SCT; the provision of tools, resources, and environmental changes that make a new behavior easier to perform. This was accomplished through mastery experiences in cooking and multiple exposures to a vegetable. Cooking also provides repetitive tactile interactions with unfamiliar tools and ingredients. Studies have shown that children who have the opportunity to touch, taste, and smell recipe ingredients, like produce, display fewer food aversions and may eat more of these ingredients (35). By participating in the preparation of six total recipes, children had the opportunity to “master” culinary skills through repetitive practice of basic cooking skills.

The *Healthy Helpers* program successfully increased children’s CL and their SE in cooking. The improvements in these two variables may predict future behavior changes surrounding children’s intake of vegetables. Similar research has connected enhanced CL and SE to self-selection of produce, improved vegetable intakes, and more home-cooking (18, 27, 36, 37). Improvements in culinary SE are also predictive of a higher frequency of engaging in cooking in the future, as it acts reciprocally to increase a person’s desire to repeat the behavior (18, 22, 28, 33). Overall, the short-term changes elicited by the *Healthy Helpers* program (i.e., increased acceptance of food with an added vegetable, increased CL and increased culinary SE) may lead to longer-term behavior changes necessary for increased vegetable intakes.

### 4.1. Study limitations

Despite the improvement in children’s SE to cook, we did not see a significant increase in children’s willingness to try vegetables. This outcome was reasonably anticipated for several reasons. The *Healthy Helpers* curriculum consisted of a six-week-long virtual intervention, and its brief timeframe may have decreased the likelihood of substantial behavioral change. While short-term programs like *Healthy Helpers* provide valuable insights, inspiration, and initial momentum, they often fall short when it comes to addressing the range of factors necessary to create significant behavioral change.

The virtual aspect of the program and the lack of in-person instruction/activities also limited our ability to ensure program fidelity. There was no way to ensure that children tasted all the recipes or that they were prepared as intended. For example, in a group discussion, one child shared that they fried the pocket pizza recipe instead of baking it. Another child claimed they did not like mushrooms and intended to leave them out entirely. Altered recipes and procedures may have limited the intervention’s ability to provide repeated exposures to mushrooms and taste testing. In fact, there was ultimately no way to ensure that the children, not their caregivers, were responsible for most of the cooking process. These factors are linked to dietary behavior change in the literature. In addition, children might be more willing to try a new food during an in-person class since they are in the presence of their peers during the cooking and tasting process. Virtual learning lacks the in-person observation and social influence/persuasion that in-person cooking classes provide. The virtual aspect of the program may also account for the limited sample sizes as we could not collect surveys in real time and many child participants did not complete or turn in study surveys.

An additional reason we may not have observed a significant increase in children’s overall willingness to try vegetables may be linked to the sensory attributes of the vegetable added to recipes (mushrooms). Numerous perceptual features have been related to a child’s acceptance or rejection of new foods, including texture, flavor/taste, aroma, and appearance (38). We may have observed little improvement in children’s acceptance of recipes and willingness to try vegetables since mushrooms were the only added vegetable in the recipes. As previously stated, some children may have disliked mushrooms’ unique characteristics, resulting in a negative response when answering the survey question regarding willingness to try vegetables. Further, since the lessons excluded any nutrition education about mushrooms, children may not have been aware that mushrooms are classified as vegetables, not as fruits or part of another food group. If children did not perceive mushrooms as a vegetable, the survey responses to measure willingness to eat vegetables might not have truly reflected their feelings. We may have observed a greater impact on the acceptability of vegetable-added recipes if recipes featured a larger variety of vegetables that provided different sensory experiences to the children.

## 5. Conclusion

Children exposed to a 6-week *Healthy Helpers* virtual culinary curriculum combined with hands-on cooking experiences accepted vegetables most readily when they were incorporated into a recipe where the vegetable was hidden and masked by familiar flavors and spices. Post-program measurements showed improvement in children’s cooking knowledge and their self-efficacy to cook. School-based nutrition programs should consider incorporating CL and hands-on cooking lessons into existing nutrition education programming. CL may be the missing piece that has prevented other programs or interventions from eliciting significant improvements in children’s vegetable intake. Dietary behavior changes often take time. Longer duration CL programs should be implemented that incorporate repeated exposure to multiple vegetables. While virtual programs have benefits, in-person CL programs are superior in ensuring process fidelity while preparing recipes and tasting finished food products. In addition, measures of children’s acceptance of tasted recipes should feature a mixed method model with qualitative testing that allows researchers to examine sensory attributes that were accepted or disliked by children. Finally, future research should include longer-term data collection to examine the sustained impacts of experiential CL programs. Involving children in cooking and improving their CL and SE to cook may be the best way to transform nutrition education into daily food practice.

## Data availability statement

The raw data supporting the conclusions of this article will be made available by the authors, without undue reservation.

## Ethics statement

The studies involving humans were approved by Rutgers University Institutional Review Board. The studies were conducted in accordance with the local legislation and institutional requirements. Written informed consent for participation in this study was provided by the participants’ legal guardians/next of kin.

## Author contributions

PP conceived and designed the research protocol including survey tools, performed data analysis, and wrote this manuscript in conjunction with other authors. AB assisted in designing the research protocol, including survey tools, submitted and received Institutional Review Board approval from Rutgers University, developed program curriculum and recipes, developed all tables and figures, and wrote this manuscript in conjunction with PP and EC. EC conceived and designed the research protocol including survey tools, assisted in program curriculum development, and wrote or edited this manuscript in conjunction with PP and AB. All authors contributed to the article and approved the submitted version.

## References

[1] Nekitsing C Blundell-Birtill P Cockroft JE Hetherington MM. Systematic review and meta-analysis of strategies to increase vegetable consumption in preschool children aged 2–5 years. Appetite. (2018) 127:138–54. doi: 10.1016/j.appet.2018.04.019, PMID: 29702128

[2] Slavin J. Dietary guidelines: are we on the right path? Nutr Today. (2012) 47:245–51. doi: 10.1097/NT.0b013e31826c50af, PMID: 37122954

[3] Snetselaar DG de Jesus JM DeSilva DM Stoody EE. Dietary guidelines for Americans, 2020–2025. Nutr Today. (2021) 56:164. doi: 10.1097/NT.0000000000000512 PMC8713704 34987271

[4] Wambogo EA Ogden CL. Fruit and vegetable consumption among children and adolescents in the United States, 2015–2018. NCHS Data Brief. (2020):8. 33270555

[5] Landry MJ Burgermaster M van den Berg AE Asigbee FM Vandyousefi S Ghaddar R. Barriers to preparing and cooking vegetables are associated with decreased home availability of vegetables in low-income households. Nutrients. (2020) 12:1823. doi: 10.3390/nu12061823, PMID: 32570923 PMC7353206

[6] Evans KR Hudson SV. Engaging the community to improve nutrition and physical activity among houses of worship. Prev Chronic Dis. (2014) 11:130270. doi: 10.5888/pcd11.130270, PMID: 24625362 PMC3958142

[7] Nepper MJ Chai W. Parents’ barriers and strategies to promote healthy eating among school-age children. Appetite. (2016) 103:157–64. doi: 10.1016/j.appet.2016.04.012, PMID: 27090341

[8] Pescud M Pettigrew S. Parents’ experiences with hiding vegetables as a strategy for improving children’s diets. Br Food J. (2014) 116:1853–63. doi: 10.1108/BFJ-06-2012-0155

[9] Ard JD Fitzpatrick S Desmond RA Sutton BS Pisu M Allison DB. The impact of cost on the availability of fruits and vegetables in the homes of schoolchildren in Birmingham, Alabama. Am J Public Health. (2007) 97:367–72. doi: 10.2105/AJPH.2005.080655, PMID: 17138914 PMC1781416

[10] Crary IL Ardoin NM Gardner C. Impact of child interaction with food preparation on vegetable preferences: a farm-based education approach. J Nutr Educ Behav. (2022) 54:46–55. doi: 10.1016/j.jneb.2021.08.009 34776345

[11] Asigbee FM Davis JN Markowitz AK Landry MJ Vandyousefi S Ghaddar R. The association between child cooking involvement in food preparation and fruit and vegetable intake in a Hispanic youth population. Curr Dev Nutr. (2020) 4:nzaa028. doi: 10.1093/cdn/nzaa028, PMID: 32258989 PMC7108796

[12] Metcalfe JJ Leonard D. Reprint of “the relationship between culinary skills and eating behaviors: challenges and opportunities for parents and families.”. Physiol Behav. (2018) 193:302–6. doi: 10.1016/j.physbeh.2018.07.006, PMID: 30099991

[13] Ford AD Colby SE McElrone M Franzen-Castle L Olfert MD Kattelmann KK. Cooking frequency associated with dietary quality in iCook-4H youth participants at baseline. Nutr Metab Insights. (2019) 12:1178638819836790. doi: 10.1177/1178638819836790, PMID: 31168293 PMC6484674

[14] Fertig AR Loth KA Trofholz AC Tate AD Miner M Neumark-Sztainer D. Compared to pre-prepared meals, fully and partly home-cooked meals in diverse families with young children are more likely to include nutritious ingredients. J Acad Nutr Diet. (2019) 119:818–30. doi: 10.1016/j.jand.2018.12.006, PMID: 30765316 PMC6487205

[15] Hartmann C Dohle S Siegrist M. Importance of cooking skills for balanced food choices. Appetite. (2013) 65:125–31. doi: 10.1016/j.appet.2013.01.016, PMID: 23402717

[16] Maiz E Urkia-Susin I Urdaneta E Allirot X. Child involvement in choosing a recipe, purchasing ingredients, and cooking at school increases willingness to try new foods and reduces food neophobia. J Nutr Educ Behav. (2021) 53:279–89. doi: 10.1016/j.jneb.2020.12.015, PMID: 33573994

[17] Fredericks L Koch PA Alicia LA Galitzdorfer L Costa A Utter J. Experiential features of culinary nutrition education that drive behavior change: frameworks for research and practice. Health Promot Pract. (2020) 21:331–5. doi: 10.1177/1524839919896787, PMID: 32011916

[18] Jarpe-Ratner E Folkens S Sharma S Daro D Edens NK. An experiential cooking and nutrition education program increases cooking self-efficacy and vegetable consumption in children in grades 3–8. J Nutr Educ Behav. (2016) 48:697–705.e1. doi: 10.1016/j.jneb.2016.07.021, PMID: 27575849

[19] Tuorila H Mustonen S. Reluctant trying of an unfamiliar food induces negative affection for the food. Appetite. (2010) 54:418–21. doi: 10.1016/j.appet.2010.01.010, PMID: 20097240

[20] Davis JN Ventura EE Cook LT Gyllenhammer LE Gatto NM. LA sprouts: a gardening, nutrition, and cooking intervention for Latino youth improves diet and reduces obesity. J Am Diet Assoc. (2011) 111:1224–30. doi: 10.1016/j.jada.2011.05.009, PMID: 21802571

[21] Walters LM Stacey JE. Focus on food: development of the cooking with kids experiential nutrition education curriculum. J Nutr Educ Behav. (2009) 41:371–3. doi: 10.1016/j.jneb.2009.01.004, PMID: 19717122

[22] Guinard JX Myrdal Miller A Mills K Wong T Lee SM Sirimuangmoon C. Consumer acceptance of dishes in which beef has been partially substituted with mushrooms and sodium has been reduced. Appetite. (2016) 105:449–59. doi: 10.1016/j.appet.2016.06.018, PMID: 27317972

[23] Myrdal Miller A Mills K Wong T Drescher G Lee SM Sirimuangmoon C. Flavor-enhancing properties of mushrooms in meat-based dishes in which sodium has been reduced and meat has been partially substituted with mushrooms: flavor-enhancing properties of mushrooms. J Food Sci. (2014) 79:S1795–804. doi: 10.1111/1750-3841.12549 25124478

[24] Landers P Shults C. Pots, pans, and kitchen equipment: Do low-income clients have adequate tools for cooking?. (2008) Available at: https://archives.joe.org/joe/2008february/rb4.php (Accessed 16 November 2022)

[25] Amin SA Lehnerd M Cash SB Economos CD Sacheck JM. Development of a tool for food literacy assessment in children (TFLAC). J Nutr Educ Behav. (2019) 51:364–9. doi: 10.1016/j.jneb.2018.12.006, PMID: 30851841

[26] Glanz K Rimer BK Viswanath K. Health behavior: Theory, research, and practice. 5th ed. San Francisco, California: Jossey-Bass (2015).

[27] Soldavini J Taillie LS Lytle LA Berner M Ward DS Ammerman A. Cooking matters for kids improves attitudes and self-efficacy related to healthy eating and cooking. J Nutr Educ Behav. (2021) 54:211–8. doi: 10.1016/j.jneb.2021.09.004 34774426

[28] Spill MK Birch LL Roe LS Rolls BJ. Hiding vegetables to reduce energy density: an effective strategy to increase children’s vegetable intake and reduce energy intake. Am J Clin Nutr. (2011) 94:735–41. doi: 10.3945/ajcn.111.015206, PMID: 21775554 PMC3155937

[29] Estay K Pan S Zhong F Capitaine C Guinard JX. A cross-cultural analysis of children’s vegetable preferences. Appetite. (2019) 142:104346. doi: 10.1016/j.appet.2019.104346, PMID: 31278955

[30] Cotton W Dudley D Peralta L Werkhoven T. The effect of teacher-delivered nutrition education programs on elementary-aged students: an updated systematic review and meta-analysis. Prev Med Rep. (2020) 20:101178. doi: 10.1016/j.pmedr.2020.101178, PMID: 32944494 PMC7481566

[31] Bergling E Pendleton D Shore E Harpin S Whitesell N Puma J. Implementation factors and teacher experience of the integrated nutrition education program: a mixed methods program evaluation. J Sch Health. (2022) 92:493–503. doi: 10.1111/josh.13153, PMID: 35174503

[32] Sutton S. Health behavior: psychosocial theories In: International Encyclopedia of the Social & Behavioral Sciences: Elsevier (2001). 6499–506.

[33] Bandura A. Social cognitive theory of self-regulation. Organ Behav Hum Decis Process. (1991) 50:248–87. doi: 10.1016/0749-5978(91)90022-L

[34] Lichtenstein AH Ludwig DS. Bring Back home economics education. JAMA J Am Med Assoc. (2010) 303:1857–8. doi: 10.1001/jama.2010.592, PMID: 20460625 PMC6886379

[35] Coulthard H Sealy A. Play with your food! Sensory play is associated with tasting of fruits and vegetables in preschool children. Appetite. (2017) 113:84–90. doi: 10.1016/j.appet.2017.02.003, PMID: 28202412

[36] Chu YL Storey KE Veugelers PJ. Involvement in meal preparation at home is associated with better diet quality among Canadian children. J Nutr Educ Behav. (2014) 46:304–8. doi: 10.1016/j.jneb.2013.10.003, PMID: 24238908

[37] Coulthard H Ahmed S. Non taste exposure techniques to increase fruit and vegetable acceptance in children: effects of task and stimulus type. Food Qual Prefer. (2017) 61:50–4. doi: 10.1016/j.foodqual.2017.04.012

[38] Pellegrino R Luckett CR. Aversive textures and their role in food rejection. J Texture Stud. (2020) 51:733–41. doi: 10.1111/jtxs.12543, PMID: 32533706

