# Supplementary material for: Healthy helpers: using culinary lessons to improve children’s culinary literacy and self-efficacy to cook
Source: Front Public Health. 2023 Nov 6;11:1156716. doi: 10.3389/fpubh.2023.1156716 (PMC10657997; doi:10.3389/fpubh.2023.1156716)
Supplement: Supplementary file 1 [file Table_1.DOCX]

**Additional Files (Tables)**

**Manuscript Title:**

Healthy Helpers: Using culinary lessons to improve children’s culinary literacy and self-efficacy to cook

| **Lesson #** | **Main Cooking Skill Concept Presented** | **Recipe for Cooking Demonstration (traditional vs. added vegetables)** |
| --- | --- | --- |
| **1** | Introduction to basic cooking vocabulary and kitchen tools | Macaroni and Cheese (traditional) |
| **2** | Introduction to Marinating | Beef Fajitas (traditional) |
| **3** | Grating | Mushroom Pocket Pizza (added vegetables) |
| **4** | Microwave Safety | Mushroom Macaroni and cheese (added vegetables) |
| **5** | Common Marinade Ingredients | Portabella Fajitas (added vegetables) |
| **6** | Using Scissors to Cut Foods | Pocket Pizza (traditional) |

Table 1. *Healthy Helpers: Culinary Literacy for Kids* Curriculum lesson order, main cooking concept covered, and cooking demonstration recipe.
