# Supplementary material for: Healthy helpers: using culinary lessons to improve children’s culinary literacy and self-efficacy to cook
Source: Front Public Health. 2023 Nov 6;11:1156716. doi: 10.3389/fpubh.2023.1156716 (PMC10657997; doi:10.3389/fpubh.2023.1156716)
Supplement: Supplementary file 2 [file Table_2.DOCX]

**Additional Files (Tables)**

**Manuscript Title:**

Healthy Helpers: Using culinary lessons to improve children’s culinary literacy and self-efficacy to cook

| **Variable** | **Percentage** |
| --- | --- |
| **Age**  **9 years old**  **10 years old**  **11 years old** | 3%  50%  47% |
| **Gender**  **Male**  **Female**  **Unknown** | 38%  59%  3% |
| **Free and Reduced School Lunch**  **Yes**  **No**  **Unknown** | 82%  15%  3% |
| **Race/Ethnicity**  **Hispanic**  **Black**  **White, not of Hispanic origin**  **Asian**  **Unknow**n | 87%  5%  3%  3%  2% |

Table 2. Demographic table for study participants (n=39). Data collected 2021.
