# Supplementary material for: Healthy helpers: using culinary lessons to improve children’s culinary literacy and self-efficacy to cook
Source: Front Public Health. 2023 Nov 6;11:1156716. doi: 10.3389/fpubh.2023.1156716 (PMC10657997; doi:10.3389/fpubh.2023.1156716)
Supplement: Supplementary file 3 [file Table_3.DOCX]

**Additional Files (Tables)**

**Manuscript Title:**

Healthy Helpers: Using culinary lessons to improve children’s culinary literacy and self-efficacy to cook

| **Recipe** | **n** | **Traditional Recipe Acceptability Score**  **Mean (SD)** | **Added Vegetable Recipe Acceptability Score**  **Mean (SD)** | **t** | **df** | **p-value*** | **Cohen’s D-effect size^1^** |
| --- | --- | --- | --- | --- | --- | --- | --- |
| **Macaroni & Cheese** | 13 | 1.90 (0.24) | 1.55 (0.30) | 3.600 | 12 | 0.004* | 0.998 |
| **Fajita** | 15 | 1.82 (0.35) | 1.57 (0.32) | 2.739 | 14 | 0.008* | 0.707 |
| **Pocket Pizza** | 10 | 1.70 (0.35) | 1.60 (0.38) | 0.712 | 9 | 0.494 | 0.225 |

Table 3. Paired t-test analyses of children’s acceptance of traditional recipes compared to added vegetable recipes cooked at home. Minimum acceptability score = 1; Maximum acceptability score =2.

*p<.05  ^1^Effect sizes (small =0.2, medium =0.5, large = 0.8)
