# Supplementary material for: Healthy helpers: using culinary lessons to improve children’s culinary literacy and self-efficacy to cook
Source: Front Public Health. 2023 Nov 6;11:1156716. doi: 10.3389/fpubh.2023.1156716 (PMC10657997; doi:10.3389/fpubh.2023.1156716)
Supplement: Supplementary file 4 [file Table_4.DOCX]

**Additional Files (Tables)**

**Manuscript Title:**

Healthy Helpers: Using culinary lessons to improve children’s culinary literacy and self-efficacy to cook

| **Variables** | **Pre-Intervention Mean (SD)** | **Post-**  **Intervention**  **Mean (SD)** | **t** | **df** | **p-value** | **Cohen’s D-effect size^1^** |
| --- | --- | --- | --- | --- | --- | --- |
| **Culinary Literacy Knowledge (CL)** | 68.31 (20.96) | 73.69 (15.50) | -1.811 | 38 | 0.078 | -.290 |
| **Self-Efficacy to cook (SE)** | 32.89 (14.04) | 80.42 (12.54) | -16.064 | 38 | <.001* | -2.572 |
| **Willingness to eat Vegetables (WV)** | 59.47 (34.48) | 58.67 (27.88) | -0.168 | 38 | 0.437 | 0.025 |
| **Total Score** | 56.89 (12.00) | 73.59 (14.93) | -8.088 | 38 | <.001* | -1.295 |

Table 4. Pre- and post-intervention paired t-test analyses of children’s culinary literacy knowledge, cooking self-efficacy, overall willingness to eat vegetables, and the total score (CL + SE + WV/3). Minimum mean score = 0; Maximum mean score =100.

*p<.05  ^1^Effect sizes (small =0.2, medium =0.5, large = 0.8)
